# Comparative Outcomes of Generic and Branded Dasatinib in Chronic-Phase Chronic Myeloid Leukemia: A Single-Center Within-Patient Analysis

**DOI:** 10.2147/JBM.S625380

**Published:** 2026-10-05

**Authors:** Anas Babiker, Elrazi A Ali, Alaa Rahhal, Rola Ghasoub, Amir Mohamed Nounou, Anas Hamad, Mohamed A Yassin

**Affiliations:** 1 National Center for Cancer Care and Research (NCCCR), Hamad Medical Corporation, Doha, Qatar; 2 Department of Hematology and Medical Oncology, Brown Cancer Center, University of Louisville, Louisville, KY, USA; 3 Department of Pharmacy, Heart Hospital, Hamad Medical Corporation, Doha, Qatar; 4 Department of Pharmacy, National Center for Cancer Care and Research (NCCCR), Hamad Medical Corporation, Doha, Qatar

**Keywords:** dasatinib, generic dasatinib, TKI, generic dasatinib chronic myeloid leukemia

## Abstract

**Background:**

Tyrosine kinase inhibitors (TKIs) have transformed chronic myeloid leukaemia (CML) from a fatal malignancy into a manageable chronic condition. Second-generation TKIs, including dasatinib, achieve faster, deeper molecular responses and overcome imatinib resistance or intolerance. However, high acquisition costs limit access, particularly in resource-constrained settings. The FDA approval of generic dasatinib in September 2024 represents a landmark step towards affordability.

**Methods:**

A retrospective, single-center, within-patient paired cohort study, paired before-after design was conducted at the National Center for Cancer Care and Research in Qatar. A 58 adults patients with confirmed chronic-phase CML (CP-CML) who transitioned from branded Sprycel^®^(dasatinib) to Daleubin^®^ (dasatinib) between January 2024 and December 2025 were included. Primary outcomes were hematologic and major molecular response rates. Secondary outcomes included adverse events, adherence, dose modifications, and toxicity-related hospitalizations. Paired t-tests and McNemar’s exact test were applied.

**Results:**

Fifty-eight patients were included (mean follow-up 6 months). Complete hematologic response was achieved (maintained) in all patients (100%); major molecular response in 56 (96.6%). No grade 3–4 adverse events occurred. Overall adverse-event rates were comparable between formulations (15.5% vs 13.8%; P = 1.000). During the follow-up period, toxicity-related hospitalizations were numerically lower with generic dasatinib but not statistically significant (1.7% vs 10.3%; P = 0.063). A statistically significant but clinically modest platelet count increase was observed post-switch (211.5 ± 64.9 vs 233.8 ± 95.4×10^9^/L; P = 0.046).

**Conclusion:**

Despite the primary limitation of a small sample size and short follow up period, generic dasatinib formulation, demonstrated short-term safety and efficacy comparable to those of the branded formulation. High response rates, favorable tolerability, and fewer hospitalizations were observed, supporting its potential as a more cost-accessible treatment option. However, these findings should be interpreted cautiously, and larger, multicenter prospective studies with longer follow-up are needed to confirm comparative effectiveness, safety, and long-term clinical outcomes.

## Introduction

Chronic Myeloid Leukemia (CML) is a type of blood cancer caused by a genetic change called the Philadelphia chromosome, which forms when there is a reciprocal translocation between chromosomes 9 and 22. This change creates the BCR::ABL1 gene, which makes a protein that causes uncontrolled growth of white blood cells.^1^ Before targeted treatments, most patients progressed to blast phase, but the discovery of tyrosine kinase inhibitors (TKIs) has greatly improved outcomes for people with CML and changed the disease entirely.^2^ Patients with chronic myeloid leukemia are expected to have normal life expectancy and improved quality of life. The current treatment goal in CML is to achieve treatment free remission.^3^ Imatinib was the first BCR::ABL1 inhibitor and transformed CML treatment after its strong results in the IRIS trial.^2^ However, many patients developed resistance to imatinib, mostly due to mutations in the kinase domain. This led to the development of second-generation drugs.^4^ Dasatinib, which blocks both BCR::ABL1 and SRC kinases, is one of the most powerful options. In laboratory studies, it is about 325 times more potent than imatinib and remains effective against most imatinib-resistant mutations, with few exceptions like T315I, F317C, F317I, F317L, F317V, and V299L.^4^ In the DASISION trial, patients who received dasatinib as their first treatment achieved faster and deeper molecular responses than those on imatinib. They had higher rates of complete cytogenetic response (CCyR) and major molecular response (MMR) at 12 months, and these benefits continued through five years of follow-up.^5^ Branded dasatinib is expensive, which is a major burden, especially in low-income countries; as TKI therapy is long-term, the ongoing cost places a heavy strain on both patients and health systems.^6^ The FDA approval of the first generic dasatinib formulation (Apotex Corp., September 2024) represents a landmark development with the potential to substantially reduce costs and improve global access to second-generation TKI therapy.^7^ Generic substitution is based on the idea that bioequivalence means clinical equivalence. While this assumption holds true for many types of drugs, it needs closer examination when it comes to targeted cancer therapies.^6^,^8^ To date, published real-world evidence regarding the clinical equivalence of generic dasatinib remains sparse. The present study was therefore conducted to evaluate the safety and efficacy of the new generic dasatinib compared with branded dasatinib in a cohort of CP-CML patients at a tertiary haemato-oncology center in Qatar.

## Methods

This was a retrospective, single-centre, within-patient paired cohort study included 58 patients conducted at the National Center for Cancer Care and Research (NCCCR), Hamad Medical Corporation in Qatar. The study employed a within-patient paired design in which each patient served as their own control, allowing direct comparison of outcomes during branded dasatinib treatment (pre-switch period) with those observed following transition to generic dasatinib (post-switch period). generic dasatinib was approved by the Ministry of Public Health (MoPH) in Qatar. The study was reported in accordance with the Strengthening the Reporting of Observational Studies in Epidemiology (STROBE) guidelines.

Eligible participants were adults aged 18 years or older with a confirmed diagnosis of CP-CML, based on European LeukemiaNet (ELN) diagnostic criteria and documented BCR::ABL1 −1 positivity by conventional cytogenetics, FISH testing, or RT-qPCR. All included patients had received branded dasatinib prior to mandatory transition to the generic formulation during the study period. A minimum exposure of three months to each formulation was required and all patients were in major molecular response. Patients were excluded if they had accelerated- or blast-phase disease, prior allogeneic hematopoietic stem cell transplantation, or incomplete clinical, laboratory, or outcome data.

Clinical and treatment-related data were extracted from institutional electronic medical records and the pharmacy database. Variables collected included demographic characteristics (age, sex, BMI, geographic origin, smoking status), Eastern Cooperative Oncology Group (ECOG) performance status, prior TKI therapy, dasatinib starting dose, dose modifications, treatment adherence, complete blood count parameters, adverse events (graded per National Cancer Institute - Common Terminology Criteria for Adverse Events v5.0), hematologic and molecular response status, and toxicity-related hospitalizations.

Primary outcomes were hematologic response (HR) and major molecular response (MMR) during the generic dasatinib period. Complete hematologic response (CHR) required normalization of peripheral blood counts and resolution of disease signs and symptoms. Major molecular response was defined per ELN criteria as BCR::ABL1  transcript levels ≤0.1% on the International Scale (IS), corresponding to MMR or deeper. Secondary outcomes included changes in hematologic parameters, adverse-event incidence and severity, toxicity-related hospitalizations, treatment adherence, and dose adjustments. All analyses were performed using IBM SPSS Statistics (version 27.0). Continuous variables are reported as mean ± SD and categorical variables as frequencies with percentages. Paired t-tests were used to compare continuous hematologic parameters before and after the formulation switch. McNemar’s exact test was applied for paired categorical outcomes.

All tests were two-sided; P < 0.05 was considered statistically significant. The study was approved by the Institutional Review Board of Hamad Medical Corporation (MRC Number: 01–25-1115). The requirement for individual informed consent was waived in accordance with the retrospective nature of the study. All patient data were fully de-identified Figure 1.

**Figure 1:**
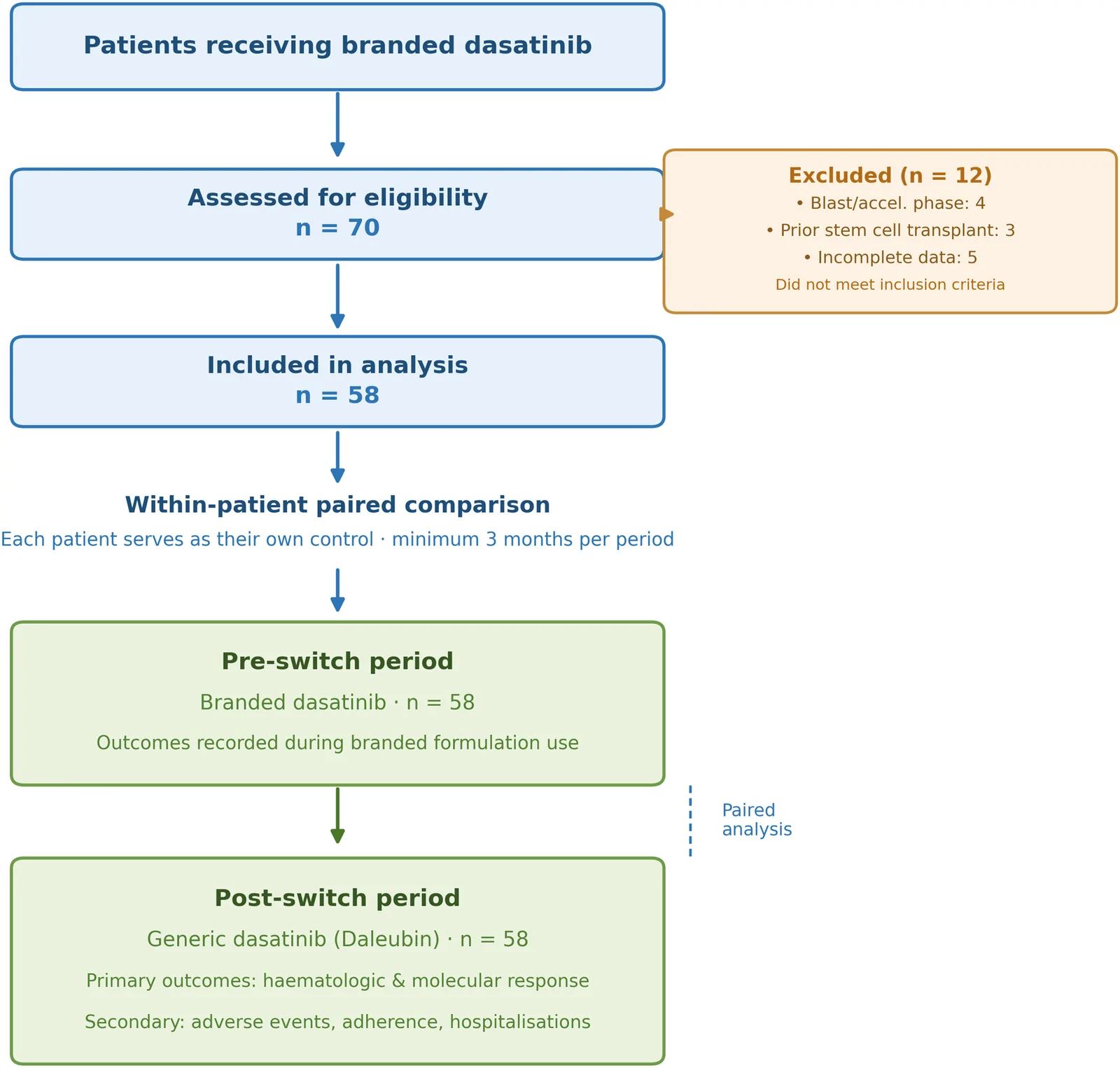
Study flow diagram. Patients receiving branded dasatinib for chronic-phase chronic myeloid leukemia (CP-CML) were assessed for eligibility, with exclusions applied before study entry, leaving 58 patients included in the final analysis. A within-patient paired design was used, in which each patient served as their own control across two sequential treatment periods separated by a minimum of 3 months each: a pre-switch period on branded dasatinib, followed by a post-switch period on generic dasatinib.

## Results

A total of 58 patients with CP-CML who transitioned from branded to generic dasatinib were included (Table 1). The cohort had a mean age of 43.2 ± 13.6 years and a mean BMI of 26.7 ± 4.1 kg/m^2^. The study population was geographically diverse, with patients predominantly from Asia (51.7%), followed by the Middle East (32.8%), Africa (12.1%), Europe (1.7%), and Australia (1.7%). Most patients were non-smokers (89.7%). There was no loss of follow-up. Regarding prior TKI exposure, 55.2% received dasatinib as front-line therapy, 31.0% had received imatinib, 8.6% nilotinib, and 5.2% sequential imatinib then nilotinib. The most common starting dose was 100 mg once daily (56.9%), followed by 50 mg once daily (43.1%) Figure 2.

**Table 1:** Baseline Characteristics of Patients Receiving Generic Dasatinib (N = 58)

Characteristic	Value (N = 58)
***Demographics***	
Age, years, mean ± SD	43.2 ± 13.6
Body mass index, kg/m^2^, mean ± SD	26.7 ± 4.1
***Geographic region*, n *(%)***	
Asia	30 (51.7)
Middle East	19 (32.8)
Africa	7 (12.1)
Europe	1 (1.7)
Australia	1 (1.7)
***Smoking status*, n *(%)***	
Non-smoker	52 (89.7)
Smoker	6 (10.3)
***ECOG performance status*, n *(%)***	
0	13 (22.4)
1	36 (62.1)
2	6 (10.3)
3	3 (5.2)
***Prior treatment*, n *(%)***	
None (first-line dasatinib)	32 (55.2)
Imatinib	18 (31.0)
Imatinib → Nilotinib	3 (5.2)
Nilotinib	5 (8.6)
***Starting dose of generic dasatinib*, n *(%)***	
100 mg once daily	33 (56.9)
50 mg once daily	25 (43.1)

**Notes**: Data are presented as mean ± standard deviation (SD) or n (%) unless otherwise indicated.

**Abbreviations**: BMI, body mass index; ECOG, Eastern Cooperative Oncology Group; SD, standard deviation.

**Figure 2:**
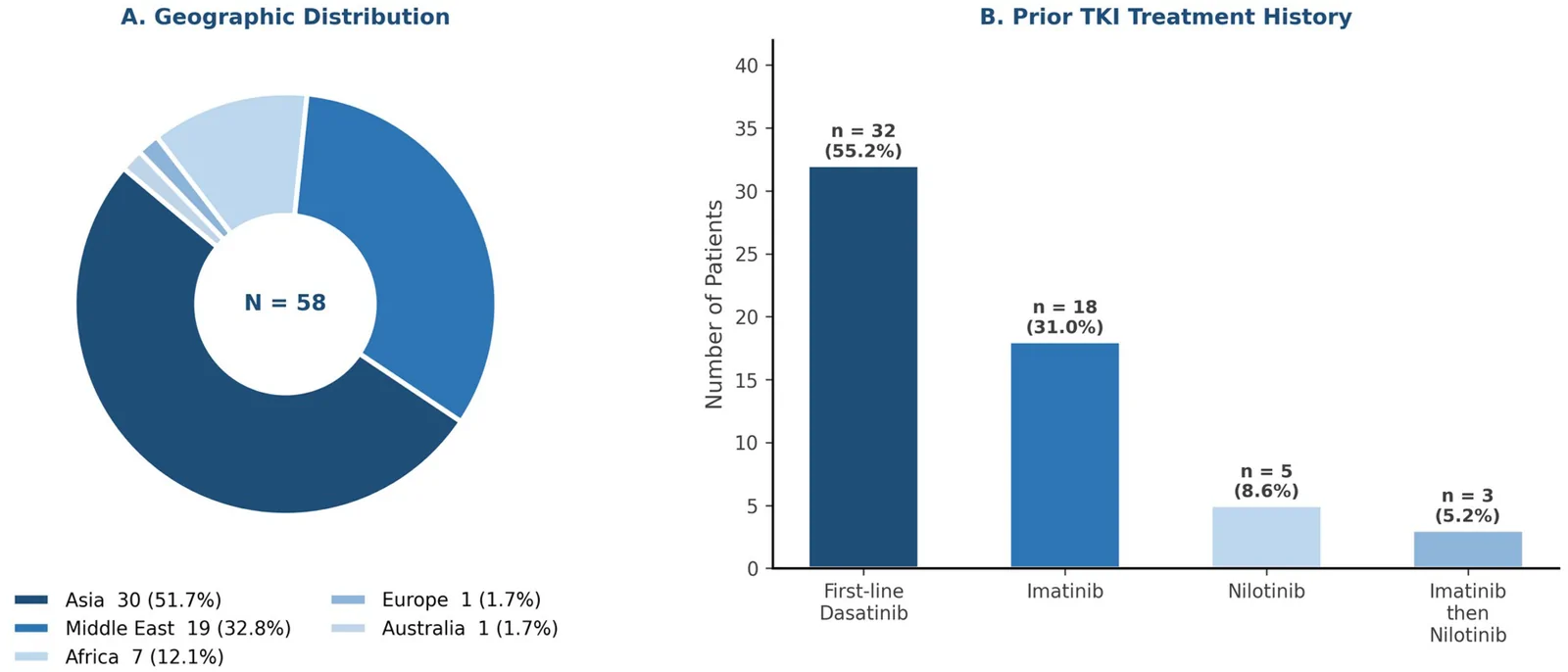
Baseline patient characteristics (N = 58). (**A**) Geographic distribution of the study cohort by region of origin: Asia (51.7%), Middle East (32.8%), Africa (12.1%), Europe (1.7%), and Australia (1.7%). (**B**) Prior tyrosine kinase inhibitor (TKI) treatment history, showing the number and percentage of patients who received dasatinib as first-line therapy (n = 32, 55.2%) versus prior imatinib (n = 18, 31.0%), nilotinib (n = 5, 8.6%), or sequential imatinib then nilotinib (n = 3, 5.2%) before study entry.

### Treatment Outcomes and Management

Treatment outcomes on generic dasatinib were highly favorable (Table 2).

**Table 2:** Treatment Outcomes and Management on Generic Dasatinib (N = 58)

Outcome	n (%)
***Efficacy outcomes***	
Hematologic response achieved	58 (100.0)
Molecular response achieved	56 (96.5)
Loss of response	2 (3.4)
***Treatment management***	
Adherence to therapy	55 (94.8)
Dose adjustments required	12 (20.7)

**Notes**: Data are presented as n (%). Outcomes refer to the on-generic-dasatinib observation period.

Complete hematologic response was mintained in all 58 patients (100%), and major molecular response in 56 patients (96.6%). Loss of response was observed in only two patients (3.4%): one patient was found to be non-compliant with their prescribed regimen, and the other was suspected of developing a BCR::ABL1 kinase domain mutation, necessitating transition to a more potent TKI. Treatment adherence was high at 94.8%. Dose adjustments were required in 12 patients (20.7%), reflecting tolerability and response optimization decisions Figure 3.

**Figure 3:**
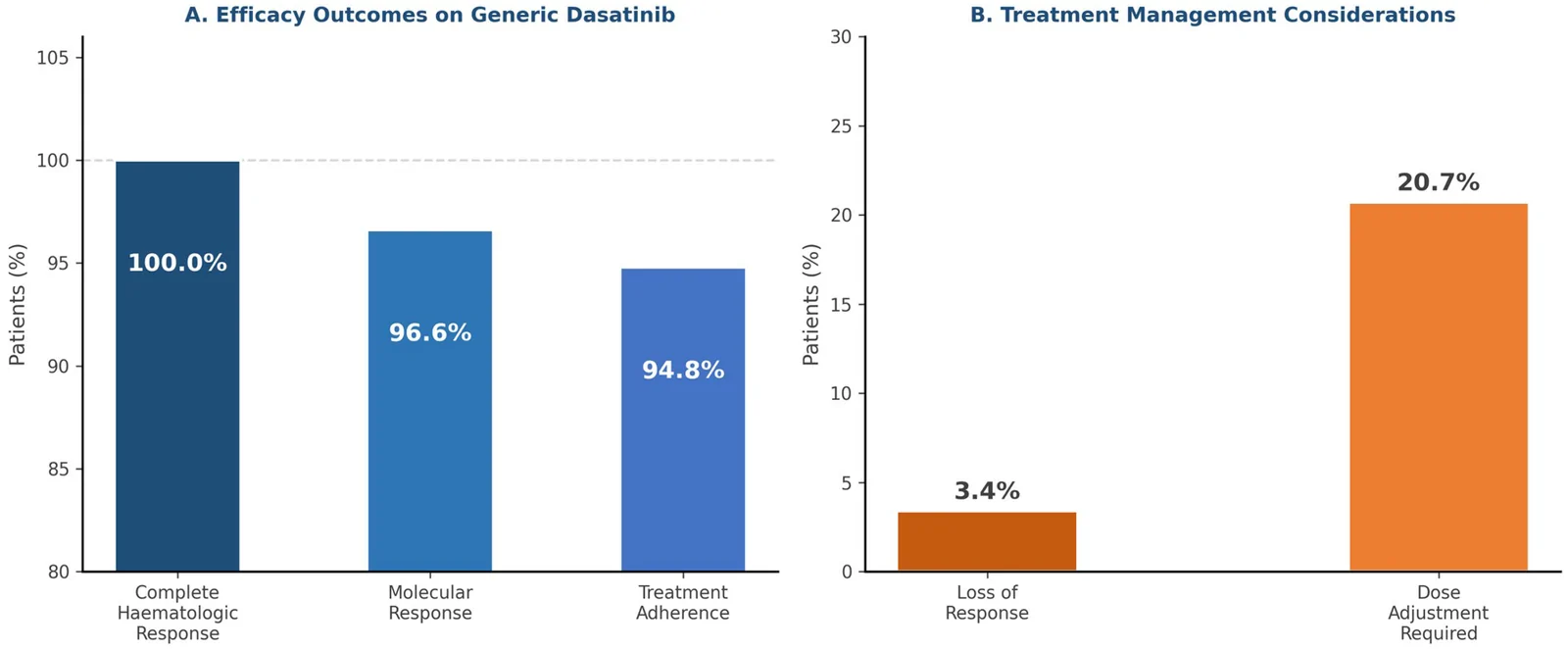
Treatment efficacy and management outcomes on generic dasatinib (N = 58). (**A**) Key efficacy endpoints achieved during the generic dasatinib treatment period, including complete haematologic response (100.0%), major molecular response (96.6%), and treatment adherence (94.8%). (**B**) Treatment management considerations, showing the proportion of patients with loss of response (3.4%) and the proportion requiring dose adjustment (20.7%) during the generic dasatinib period.

### Safety and Adverse Events

Generic dasatinib was generally well tolerated (Table 3).

**Table 3:** Comparison of Adverse Events Between Branded and Generic Dasatinib (N = 58)

Adverse Event	Branded Dasatinib(Pre-Switch) n (%)	Generic Dasatinib(Post-Switch) n (%)	P value
No adverse event	49 (84.5)	50 (86.2)	1.000
Pleural effusion	1 (1.7)	3 (5.2)	0.618
Cytopenia	5 (8.6)	1 (1.7)	0.206
Gastrointestinal bleeding	2 (3.4)	0 (0.0)	0.496
Heart failure	1 (1.7)	0 (0.0)	1.000
Intolerance	0 (0.0)	2 (3.4)	0.496
Gynecomastia	0 (0.0)	1 (1.7)	1.000
Intolerance with pancytopenia	0 (0.0)	1 (1.7)	1.000
Any adverse event	9 (15.5)	8 (13.8)	1.000

**Notes**: Data are presented as n (%). P values were calculated using Fisher’s exact test comparing the proportion of patients experiencing each adverse event during the branded (pre-switch) versus generic (post-switch) observation periods. No statistically significant differences were observed for any individual adverse event or for the overall adverse-event rate.

The majority of patients (86.2%) experienced no adverse event during the post-switch period, compared with 84.5% during the branded period. Toxicities on generic dasatinib included pleural effusion (5.2%), drug intolerance (3.4%), cytopenia (1.7%), gynecomastia (1.7%), and intolerance with pancytopenia (1.7%). No grade 3 or 4 adverse events were reported in either period, Figure 4.

**Figure 4:**
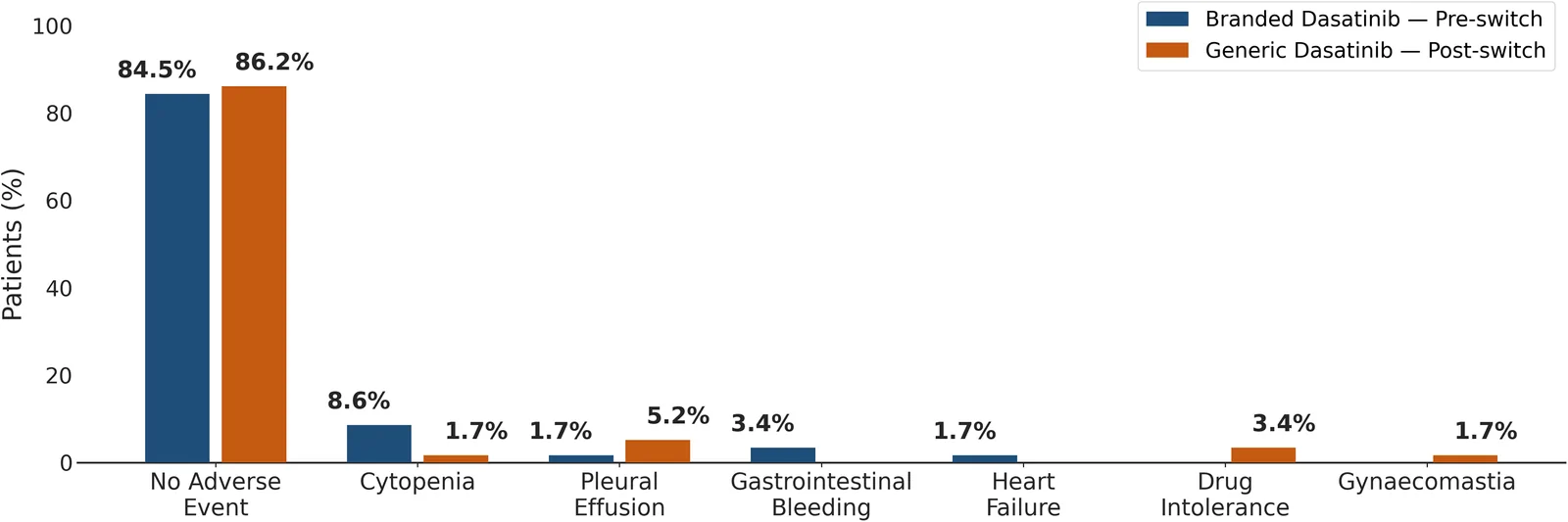
Adverse event rates with branded versus generic dasatinib. The figure displays the percentage of patients experiencing each adverse event during treatment with branded dasatinib and generic dasatinib. No statistically significant differences were observed between the two formulations for any individual toxicity (all P > 0.05, Fisher’s exact test). No grade 3–4 adverse events were reported in either group.

Comparative analysis using Fisher’s exact test revealed no statistically significant differences for any individual toxicity (all *P* > 0.05). The overall adverse-event rate was 15.5% during branded versus 13.8% during generic dasatinib (*P* = 1.000).

### Hematologic Parameters

Paired analysis of hematologic parameters revealed no clinically meaningful changes (Table 4).

**Table 4:** Comparison of haematologic parameters Before and After Switching to Generic Dasatinib (N = 58)

Parameter	Pre-Switch (Mean ± SD)	Post-Switch (Mean ± SD)	Mean Difference (95% CI)	P value
WBC count (×10^9^/L)	6.26 ± 1.84	7.33 ± 4.41	−1.07 (−2.18 to 0.04)	0.058
Platelet count (×10^9^/L)	211.5 ± 64.9	233.8 ± 95.4	−22.33 (−44.22 to −0.43)	0.046

**Notes**: Mean differences are calculated as pre-switch minus post-switch values; negative valuesindicatehigher post-switch levels. P values were derived from paired-samples t-tests.

**Abbreviations**: CI, confidence interval; SD, standard deviation; WBC, white blood cell count.

The mean WBC count showed a non-significant numerical increase from 6.26 ± 1.84 pre-switch to 7.33 ± 4.41×10^9^/L post-switch (mean difference −1.07, 95% CI −2.18 to 0.04; *P* = 0.058). The platelet count demonstrated a statistically significant increase from 211.5 ± 64.9 to 233.8 ± 95.4×10^9^/L (mean difference −22.33, 95% CI −44.22 to −0.43; *P* = 0.046). This change, whilst statistically significant, remained within the normal physiological range and is not considered clinically relevant.

### Toxicity-Related Hospitalizations

Hospitalizations attributable to drug toxicity were numerically less frequent during the generic dasatinib period (Table 5).

**Table 5:** Hospitalizations due to Toxicity Before and After Switching to Generic Dasatinib (N = 58)

Outcome	Branded Dasatinib(Pre-Switch) n (%)	Generic Dasatinib(Post-Switch) n (%)	P value
Hospitalizations due to toxicity	6 (10.3)	1 (1.7)	0.0625

**Notes**: Data are presented as n (%). The P value was calculated using McNemar’s exact test for paired binary data, comparing the proportion of patients hospitalised due to toxicity during the branded (pre-switch) versus generic (post-switch) observation periods. P-value obtained through McNemar test; There was a numerical reduction in hospitalization due to toxicity after switching to generic dasatinib (10.3% vs 1.7%, P= 0.0625).

Six patients (10.3%) were hospitalised due to toxicity while on branded dasatinib, compared with only one patient (1.7%) during the generic dasatinib period. This 83% relative reduction did not reach statistical significance (P = 0.063, McNemar’s exact test), likely reflecting the limited sample size. This finding is interpreted as a clinically meaningful trend warranting prospective evaluation in larger cohorts Figure 5.

**Figure 5:**
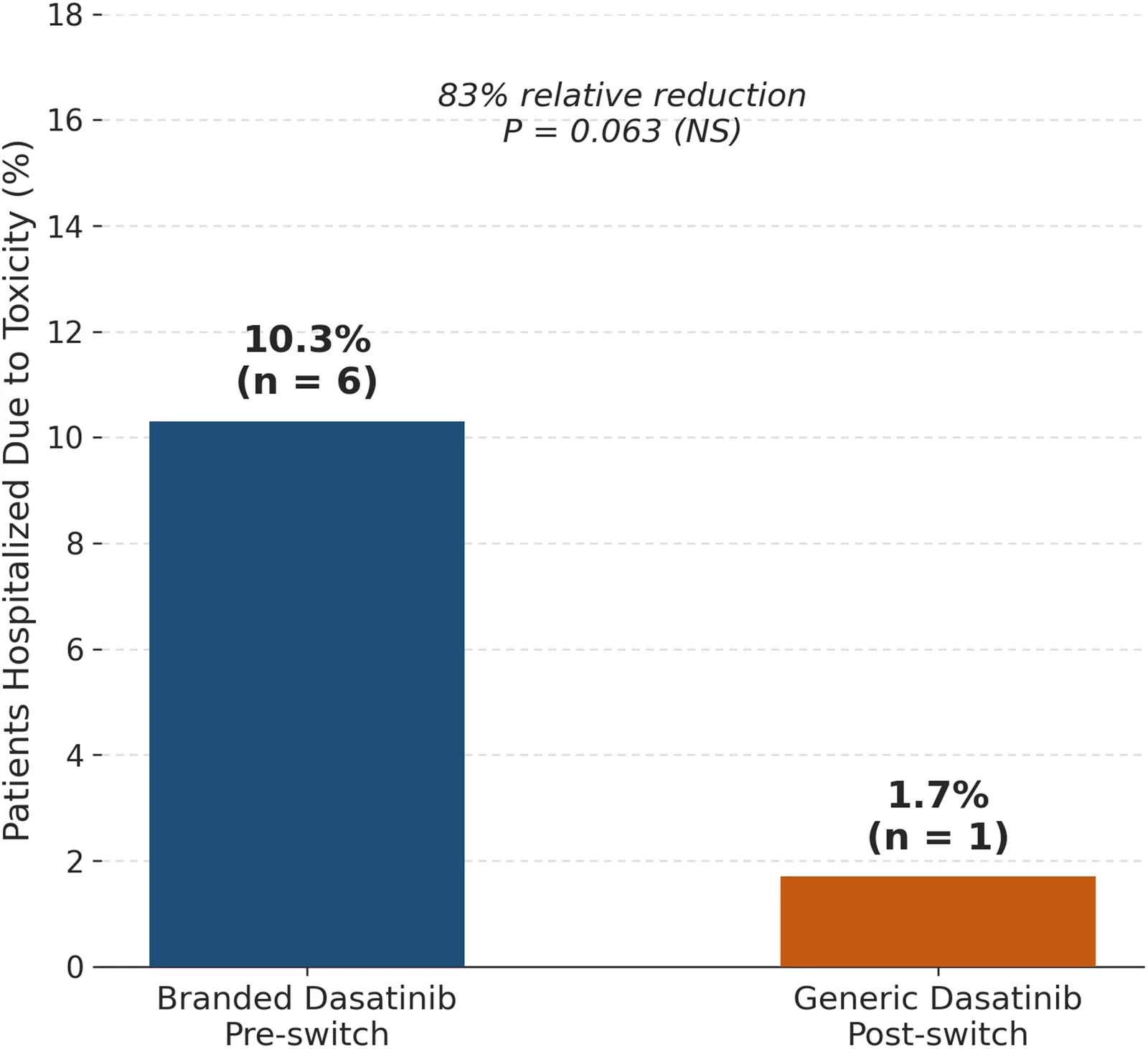
Toxicity-related hospitalizations: branded versus generic dasatinib (N = 58). Proportion of patients hospitalised due to drug-related toxicity during the branded dasatinib (pre-switch) period compared with the generic dasatinib (post-switch) period, using McNemar’s exact test. Hospitalizations occurred in 6 patients (10.3%) during the branded period versus 1 patient (1.7%) during the generic period, an 83% relative reduction; this difference did not reach statistical significance (P = 0.063).

## Discussion

With the introduction of tyrosine kinase inhibitors (TKIs), the management of chronic myeloid leukemia (CML) has improved dramatically,^9^ with patients now achieving near-normal life expectancy and an increased focus on quality of life.^10–13^ Currently, non–CML-related causes, particularly cardiovascular disease, have emerged as a leading cause of mortality in this population.^14^ The new goal of therapy is to achieve treatment-free remission after the required treatment milestones have been reached. One obstacle to this goal is limited access to second-generation TKIs. Dasatinib was first approved by the FDA in 2006 for patients with imatinib resistance or intolerance, and its indication was later expanded in 2010 to include newly diagnosed chronic-phase Ph+ CML. The first generic formulation of dasatinib became commercially available in the United States, launched by Apotex Corp. in September 2024.^7^ Since the first commercial availability of generic dasatinib, little data are available regarding its safety and efficacy in clinical practice. Approval of generic medications is based on pharmacokinetic bioequivalence studies, which are usually conducted in healthy adult volunteers under controlled conditions; these findings do not always extrapolate reliably to oncology patients, While demonstrating bioequivalence is a necessary step, it may not be sufficient to confirm that the drug performs the same way in CML patients. In CML, the goal is not merely to reach adequate drug levels in the blood, but to achieve and maintain a deep molecular response over many years, with treatment-free remission as the ultimate objective.^8^,^15^ Also, differences in formulation, such as the type of excipients used, tablet hardness, and how the tablet dissolves in the lab, can affect how the drug is absorbed. These effects may not show up in single-dose crossover studies, especially in patients with digestive problems, those taking other medications, or those with irregular eating habits.^16^ Little data exist about generic dasatinib; one study evaluated the generic dasatinib in patients with newly diagnosed CML.^17^ The study included 55 patients, and it showed that generic dasatinib is effective in achieving major molecular response (MMR). A randomized controlled trial compared low-dose generic dasatinib with imatinib, demonstrating that low-dose generic dasatinib (50 mg/day) was well tolerated and associated with an earlier and deeper molecular response rate compared with imatinib (400 mg/day).^18^ Our study is the first study to compare the switch for branded dasatinib to generic dasatinib. The present study provides, to our knowledge, the first within-patient paired comparison of generic dasatinib versus the branded dasatinib in patients with CP-CML. The principal finding is that transition from branded to generic dasatinib was associated with preserved hematologic and major molecular response rates approaching 100%, a safety profile statistically comparable to that of the originator product, high treatment adherence, and a clinically meaningful trend towards fewer toxicity-related hospitalizations.

Patient adherence is a critical factor necessary to improve molecular outcomes in CML. The ADAGIO study^6^ and later analyses^19^,^20^ showed that patients who took less than 85–90% of their prescribed imatinib doses were much less likely to maintain CCyR, and the same threshold probably applies to second-generation TKIs. Switching from one formulation to another can create uncertainty for patients, and concerns about whether generics are truly equivalent, whether justified or not, have been shown to affect how regularly patients take their medication. On the other hand, the lower cost of generics may help reduce treatment interruptions in patients who previously struggled to afford their medication.^21^ The safety profile of dasatinib also deserves attention. Pleural effusion is the most distinctive side effect, occurring in up to 28% of patients during long-term follow-up. It is managed by stopping the drug temporarily, giving diuretics, or using corticosteroids, depending on its severity.^22^ Pulmonary arterial hypertension is less common but is a serious complication of this drug.^23^ Bone marrow suppression, which includes neutropenia, thrombocytopenia, and anemia, is also a known side effect and may require dose adjustment.

One of the great advantages of generic dasatinib is the low cost. After the presence of the standard dasatinib in the market for over 10 years, the generic dasatinib provides a more affordable, identical alternative that can be available to a broader population. This translates into improved treatment initiation and adherence, particularly in resource-limited settings, where financial barriers often delay or limit access to tyrosine kinase inhibitors. In addition, reduced drug costs will decrease the overall financial constraint associated with long-term therapy, which is especially relevant in chronic conditions such as chronic myeloid leukemia that require continuous treatment. On the other hand, the use of generic dasatinib can significantly reduce overall expenditure, allowing for better allocation of resources. As bioactivity and safety are maintained, the clinical outcomes should be equivalent to those achieved with the brand dasatinib. However, ongoing real-world data are essential to confirm long-term effectiveness and safety across different patient populations.

The data showed that there are no statistically significant differences observed in the rates of adverse events between the standard and the generic dasatinib, with all p-values >0.05. However, interpreting the finding should be done with caution, as there was a significant difference in the duration of use between the two formulations. However, the data suggest that both have comparable safety profiles. Cytopenia and toxicity-related hospitalizations were more frequent in the standard dasatinib group (8.6% vs 1.7% and 10.3% vs 1.7%, respectively), suggesting a potential trend toward improved tolerability with the generic formulation. However, we had to consider that adverse events that usually occur early with the original dasatinib may have already stabilized prior to the switch and contribute to lower toxicity related hospitalization in generic dasatinib Conversely, pleural effusion appeared slightly higher in the generic group (5.2% vs 1.7%), although this difference was small and not statistically significant. Other adverse events, including gastrointestinal bleeding, heart failure, and gynecomastia, were rare in both groups (p-values >0.05). The observed similarity in adverse event rates aligns with existing evidence supporting the short term effectiveness and clinical safety of generic tyrosine kinase inhibitors.

One of the most clinically meaningful findings of this study is the remarkable preservation of treatment responses following the transition from branded to generic dasatinib. All 58 patients maintained a complete hematological response on generic dasatinib and major molecular response was achieved or sustained in 96.6% of the cohort. These results are consistent with the growing body of evidence suggesting that, for the majority of CML patients in chronic phase, generic TKI formulations are capable of delivering response rates comparable to their originator counterparts.^24–26^ The near-identical response outcomes observed across both formulations in this within-patient paired design are particularly reassuring, given that each patient served as their own control, effectively eliminating the confounding influence of baseline disease characteristics, prior treatment history, and individual pharmacogenomic variability. Nevertheless, two patients experienced loss of molecular response following the switch to generic dasatinib, representing a rate of 3.4% in this cohort, one patient was non compliance to his medications, and the other due to suspicion of mutation, so shifted to another potent TKI. While this is a small number, it should not be dismissed as clinically inconsequential. Loss of molecular response in CML — even in a single patient — carries significant implications, as it may signal the emergence of BCR::ABL1 kinase domain mutations, clonal evolution, or subtherapeutic drug exposure, any of which can herald progression if not promptly identified and managed.^15^,^27^,^28^ Whether this event was attributable to a formulation-related difference in bioavailability, an episode of suboptimal adherence coinciding with the switch, or disease-intrinsic factors cannot be determined from the retrospective data available. However, the temporal association with the transition to generic dasatinib makes it a finding that warrants prospective monitoring in future studies. It is also worth noting that the two formulations appeared broadly equivalent in terms of myelosuppression, with white blood cell counts showing no statistically significant difference before and after the switch (p = 0.058). Platelet counts, however, demonstrated a statistically significant increase following the transition to generic dasatinib (p = 0.046), which could reflect subtle differences in the myelosuppressive potency between formulations, improved tolerability of the generic agent, or simply a temporal effect reflecting cumulative disease control over a longer treatment duration. This finding aligns with observations from generic imatinib studies, in which hematological parameters were generally stable or showed modest improvements following substitution, providing an additional layer of reassurance regarding the safety of generic dasatinib at the hematological level.^29^,^30^

However, these findings should be interpreted with caution given the small sample size and the modest increase in platelet count, which may not be clinically significant. Additionally, longer follow-up is needed to determine the clinical relevance and durability of this finding.

## Limitations

The principal limitation of this study is its small sample size, which limits statistical power to detect small but differences in efficacy, safety, and tolerability between standard and generic dasatinib. Consequently, the absence of statistically significant differences should be interpreted cautiously and should not be considered definitive evidence of therapeutic equivalence. The limited cohort also restricts the ability to perform meaningful subgroup analyses, and may limit the generalizability of the findings to broader and more diverse patient populations. A second important limitation is the relatively short duration of follow-up. Although early MMR and short-term safety outcomes are informative, chronic myeloid leukemia requires prolonged treatment and monitoring. Longer follow-up is needed to evaluate the durability of cytogenetic and major molecular responses, rates of treatment failure or disease progression, long-term survival outcomes, treatment adherence and discontinuation, and delayed toxicities. Extended observation is particularly important for dasatinib because adverse events such as pleural effusion and pulmonary arterial hypertension may occur after prolonged exposure. Larger, prospective studies with longer follow-up and standardized molecular monitoring are therefore warranted to more reliably establish the long-term efficacy, safety, and clinical interchangeability of generic dasatinib relative to the standard formulation. Like any other retrospective study, under-reporting bias might occur. Having the same population might lead to Carryover effects and late effects due to a lack of a washout period and. Also, our study lacks randomization and may be influenced by changes in supportive care or clinical status over time, limiting causal inference and generalizability. However, using the same patients as a control add an advantage of reducing the impact of fixed confounders and reduce the need for a larger sample size.

## Conclusion

Despite the major limitations, the data showed that generic dasatinib maintains high rates of hematologic and molecular response in the short terms, comparable to those observed with the original formulation. Overall, these findings support generic dasatinib as an effective alternative to the branded formulation, which will provide the advantage of overcoming financial barriers and expand the use of second-generation TKI, allowing patients to achieve treatment milestones. Larger prospective studies with longer follow-up are needed to confirm long-term efficacy, safety, and generalizability across broader patient populations.

## Data Availability

The data that support the findings of this study are available from the corresponding author upon reasonable request, subject to institutional data governance and patient confidentiality requirements.
